# Expression of Metallothioneins in Cutaneous Squamous Cell Carcinoma and Actinic Keratosis

**DOI:** 10.1007/s12253-012-9513-0

**Published:** 2012-03-10

**Authors:** Aleksandra Zamirska, Łukasz Matusiak, Piotr Dziegiel, Grażyna Szybejko-Machaj, Jacek C. Szepietowski

**Affiliations:** 1 Department of Dermatology, Venereology and Allergology, Wroclaw Medical University, Chałubińskiego 1, 50-368 Wrocław, Poland; 2 Department of Histology and Embryology, Wroclaw Medical University, Wroclaw, Poland; 3 Department of Histology and Embryology, Poznan Medical University, Poznan, Poland

**Keywords:** Metallothioneins, Squamous cell carcinoma, Actinic keratosis, Ki-67, Skin

## Abstract

Metallothioneins (MT) are low-molecular weight proteins implicated in heavy metal detoxification, zinc and cooper homeostasis and cell protection against free radicals. In variety of cancers MT-overexpression was shown, but there are just a few studies on the role of MT in skin carcinogenesis. Current study was undertaken to evaluate MT and Ki-67 expression in pre-cancerous skin lesions as well as in fully developed skin cancers. 73 squamous cell carcinomas (SCC), 23 actinic keratoses (AK) and 20 normal skin samples were included in the study. In obtained paraffin sections immunohistochemical reactions were performed. MT-expression in SCC (mean 2.89 ± 1.83) was significantly higher than in AK (mean 1.69 ± 1.26)(*p* = 0.006) and higher than in normal skin (mean 2 ± 0.79) (*p* = 0.0075). The MT-expression positively correlated with Ki-67 expression (*R* = 0.28; *p* = 0.017) in SCC and in AK (*R* = 0.49; *p* = 0.018). Various clinico-pathological variables, e.g. morphology, size of lesions and the depth of neoplastic infiltration were not associated to MT-expression in both SCC and AK. The grade of histological differentiation of SCC correlated positively with Ki-67 antigen (*p* < 0.001) and did not correlate with MT-expression (*p* = 0.06). Ki-67 expression was higher in SCC and in AK than in healthy skin (*p* = 0,003). In SCC and in AK expression of Ki-67 antigen correlated positively with MT-expression (respectively *p* = 0.017 and *p* = 0.018). MT may serve as a good markers of proliferation in SCC and AK. MT-overexpression in SCC may suggest a potential role of MT in skin carcinogenesis.

## Introduction

Metallothioneins (MT I-IV), small cysteine-rich, metal-binding proteins, have been described in number of organs, including the skin [1, 2]. High levels of MT-I and II, the most widely expressed isoforms, are found in liver, kidneys and skin, MT-III is predominantly expressed in brain, while MT-IV has been identified in squamous epithelia of alimentary tract [3–5]. In stratified squamous epithelia MT I and II were expressed in the basal layer, whereas MT IV was discovered in the differentiating spinous layer [5].

The main function of MT is their contribution in zinc and copper homeostasis in cells and tissues. These metallic ions, being the part of proteins and enzymes (e.g. zinc-dependent transcription factors or p53 protein), are crucial for proper cell metabolism and differentiation [2, 6]. Although, MT serve as antioxidants and protect cells’ structures against free radicals and toxic influence of heavy metal ions (Cd, Pb, Ni, Hg) [7, 8], they also have proliferative, antiapoptotic and angiogenic potential, what explains their role in oncogenesis and tumor progression [2, 9–11]. MT overexpression was revealed in variety of human tumors. In many of them a positive correlation between MT overexpression and aggressive clinical behaviour as well as poorer prognosis has been found (e.g. for carcinomas of urinary and digestive tract, breast cancers, lung carcinomas, squamous cell carcinomas of oral cavity and larynx as well as malignant melanoma) [1, 12–21]. Some researchers [15, 17, 18] suggested that less differentiated, anaplastic tumor cells were linked to higher MT expression, indicating for MT as a potential and important prognostic factor. Moreover, it was also proved that the overexpression of MT in tumor cells was responsible for the development of resistance to anticancer drugs and radiotherapy [9, 22].

Excessive exposure to ultraviolet radiation (UV) plays the main role in etiopathogenesis of actinic keratosis (AK) and squamous cell carcinoma (SCC) [23, 24]. UV causes increase in reactive oxidative species (ROS) leading to DNA damage [25]. Due to high level of free radicals, keratinocytes enhance synthesis of endogenous antioxidants, including MT. The role and behaviour of MT in skin carcnogenesis still remain unclear. It should be kept in mind, that AK being a part of a multi-step carcinogenesis process represents an early stage in a continuum that leads from carcinoma in situ to invasive SCC [26–28]. Therefore, the current study was undertaken to evaluate MT expression in SCC as well as in AK. The aim of the study was also to estimate the eventual relationships between MT expression and the expression of Ki-67 antigen as well as some clinical parameters, including the type of the tumor.

## Materials and Methods

The material consisted of 96 fair-skin biopsies obtained from 73 patients with SCC (mean age of 73.6 ± 14.2 years) and 23 subjects suffering from AK (mean age of 71.4 ± 11.2 years). The mean duration of the disease was assessed as 23.0 ± 42.4 months for SCC (range, 1–240 months) and 27.6 ± 17.7 months for AK (range, 6–84 months). The characteristics of the studied group is given in the Tables 1. Twenty samples of clinically unchanged, but sun-exposed skin, taken from healthy volunteers (mean age of 57.3 ± 18.5 years) during plastic surgery procedures, served as a control. The study was approved by the Ethics Committee of Wroclaw Medical University (protocol number-KB 609/2007).

**Table 1:** The characteristics of the studied group

Clinical parameters		Number of patients with SCC	%	Number of patients with AK	%
Age	< 60 years	8	11	4	17
	60–80 years	43	59	15	66
	> 80 years	22	30	4	17
Gender	women	29	40	18	78
	men	44	60	5	22
Size	< 2 cm	46	63	19	83
	2–5 cm	23	31,5	4	17
	> 5 cm	4	5,5	0	0
Localization	Face	39	53	14	61
	Lip	13	18	2	9
	Ear	12	16	1	9
	Scalp	2	3	0	0
	Trunk	0	0	1	4
	Upper limb	2	3	0	0
	Lower limb	5	7	5	22
Morphology	Hyperkeratotic	50	68	15	65
	Exulcerans	22	30	-	-
	Vegetans	1	2	-	-
	Erythematous	-	-	8	35

Tissue samples were immersed in 10 % buffered formalin and embedded in paraffin blocks. Alternate sections were stained with hematoxylin and eosin and used for further histological analysis. Two of us, with experience in dermatopathology, reviewed all the specimens independently (AZ and GSM). The histological grade of malignancy in SCC samples was determined using the Broders' classification based on the degree of differentiation and keratinization of tumor cells [29]. Moreover, for each tumor the degree of keratinization, nuclear polimorphism, the invasion level after Clark [30], density and depth of lymphocytes infiltration were evaluated and graded as well (Table 2). The samples of AK were devided in 3 groups taking under consideration the histological features (1 bowenoid, 8 hypertrophic and 14 of atrophic types).

**Table 2:** The histological parameters of evaluated SCC

Histological parameters		Number of patients with SCC	%
Broders	G1 (>75 % of keratinized cells)	14	19
	G2 (50–75 % of keratinized cells)	19	26
	G3 (25–50 % of keratinized cells)	25	34
	G4 (< 25 % of keratinized cells)	15	21
Depth of neoplastic infiltration according Clark scale	I- invasion into the epidermis	0	0
	II- invasion into the papillary dermis	1	1
	III- invasion to the junction of the papillary and reticular dermis	4	5
	IV- invasion into the reticular dermis	29	40
	V- invasion into the subcutaneous fat	39	54
Density of inflammatory infiltration around SCC	Mild inflammatory infiltration	19	26
	Moderate inflammatory infiltration	40	55
	Massive inflammatory infiltration	14	19
Depth of inflammatory infiltration	Dermis	18	25
	Subcutis	55	75

### Immunochemistry

Immunohistochemistry was carried out with the monoclonal antibody E9 (code M0639), which binds specifically MT-I and MT-II, using a 1:100 dilution for MT and the monoclonal antibody MIB-1 (code M7240) diluted 1:50 for Ki-67 antigen (Dako, Denmark). Four-micrometer-thick tissue sections were deparaffinized by xylene bath followed by bath with 100 %, 96 % and 70 % ethanol for 5 min. Slides were then pretreated by heating citrate buffer in a steamer and then cooled for 15 min. To block endogenous peroxidase acitivity 3 % H_2_O_2_ was added. Next the slides were rinsed in PBS for 5 min. Afterwards the primary antibody was added and slides were incubated 18 h at 4°C and washed in 3 changes of PBS. The sections were then incubated with biotinylated anti-mouse immunoglobulin for 20 min, washed again in 3 changes of PBS before being incubated with streptavidin–peroxidase complex for 20 min. The slides were visualized with diaminobenzidine chromogen (DAB) for 5 min. Finally they were placed into distilled water, stained with hematoxylin and closed with coverslips.

Slides were assessed in magnification of 100x and 200x and digitally imaged. For the evaluation of the MT expression semiquantitative Remmele scale was used (Table 3) [31]. The method takes into account both the intensity of the color reaction and the percentage of positive cells in each lesion. The final score (IRS) represents the product of these 2 values, ranging from 0 to 12 points: no reaction 0 pts., weak reaction 1–2 pts., moderate reaction 3–4 pts., intense reaction 6–12 pts. Expression of Ki-67 was also evaluated semiquantitatively by percent of immunopositive cells including the following intervals: 0 % (0 pts.), 1-10 % (1 pts.), 11–25 %, (2 pts.), 26–50 % (3 pts.) and above 50 % (4 pts.). The localization and distribution of staining within the normal skin, in AK and SCC has been studied as well.

**Table 3:** Immunoreactive Score (IRS) according to Remmele [31]

Percentage of positive cells	Intensity of stain
0 No positive cells	0 No detectable stain
1 <10 %	1 Weak nuclear stain
2 11–50 %	2 Moderate nuclear stain
3 51–80 %	3 Strong nuclear stain
4 >80 %	

### Statistical Analysis

All data were analyzed statistically with Statistica® 7.1 software (Statsoft, Cracow, Poland). Student’s *t*-test for independent variables, Mann–Whitney *U* test, univariate analysis of variance with *post hoc* test and *χ2* test were used where appropriate. Correlations between parameters were measured with Spearman’s rank correlation test. Results with p-values less than 0.05 were treated as statistically significant.

## Results

**Fig. 1:**
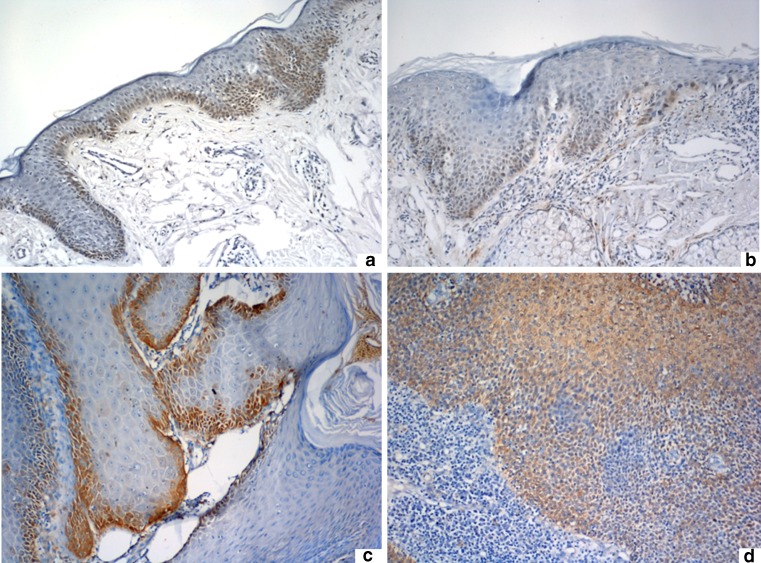
Normal, unchanged skin **a** and AK lesion **b**—MT-immunoreactivity expressed mainly in basal and parabasal layers of epithelium. Well-differentiated SCC (G2) **c** revealed peripheral MT-immunostaining, whereas poorly differentiated SCC (G4) **d**—the dispersed MT-expression. A total magnification of 100x

All the healthy skin sections, 21 cases out of AKs (92 %) and 69 cases out of SCCs (95 %) showed MT-immunoreactivity. Significant differences were observed in localization of MT-positive cells in normal skin, in AK and in SCC (*p* < 0,01). In healthy skin MT-expression was revealed in epidermal basal layer, hair matrix cells and outer hair root sheath, some cells of sebaceous and eccrine glands. In AK, MT-positive cells were found mostly in basal and parabasal layers of atypical epidermis (83 % AK). In well-differentiated SCCs (Broders 1–2) expression of MT was found in the periphery of tumor nest (61 % of cases), while in poorly differentiated SCCs (Broders 3–4) MT-positive cells were dispersed throughout the entire tumors (67.5 % cases) (Fig. 1a-d).

**Fig. 2:**
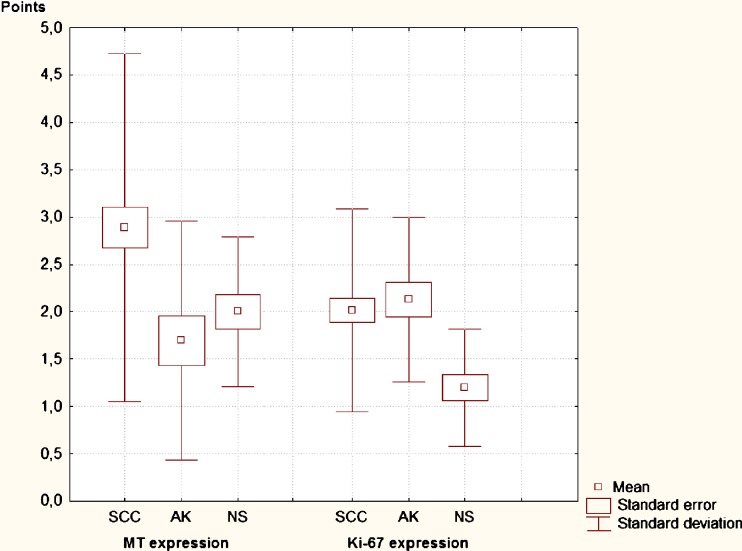
The expression of MT and Ki-67 in SCC, AK and normal (unchanged) skin. SCC—squamous cell carcinoma, AK—actinic keratosis, MT—metallothionein

The MT expression in SCC was significantly higher than for AK (*p* = 0.006) and higher than in normal skin (*p* = 0.07); no differences in MT-expression between AK and normal skin were found (*p* = 0.81) (Fig. 2.)

Ki-67 antigen was detected in 18 cases out of normal skin (90 %) and in all samples of SCC and AK. With regard to its immunostaging, the Ki-67 antigen was significantly higher expressed in SCC and in AK (with no difference between SCC and AK) than in healthy skin (*p* = 0.0025) (Fig. 2).

**Fig. 3:**
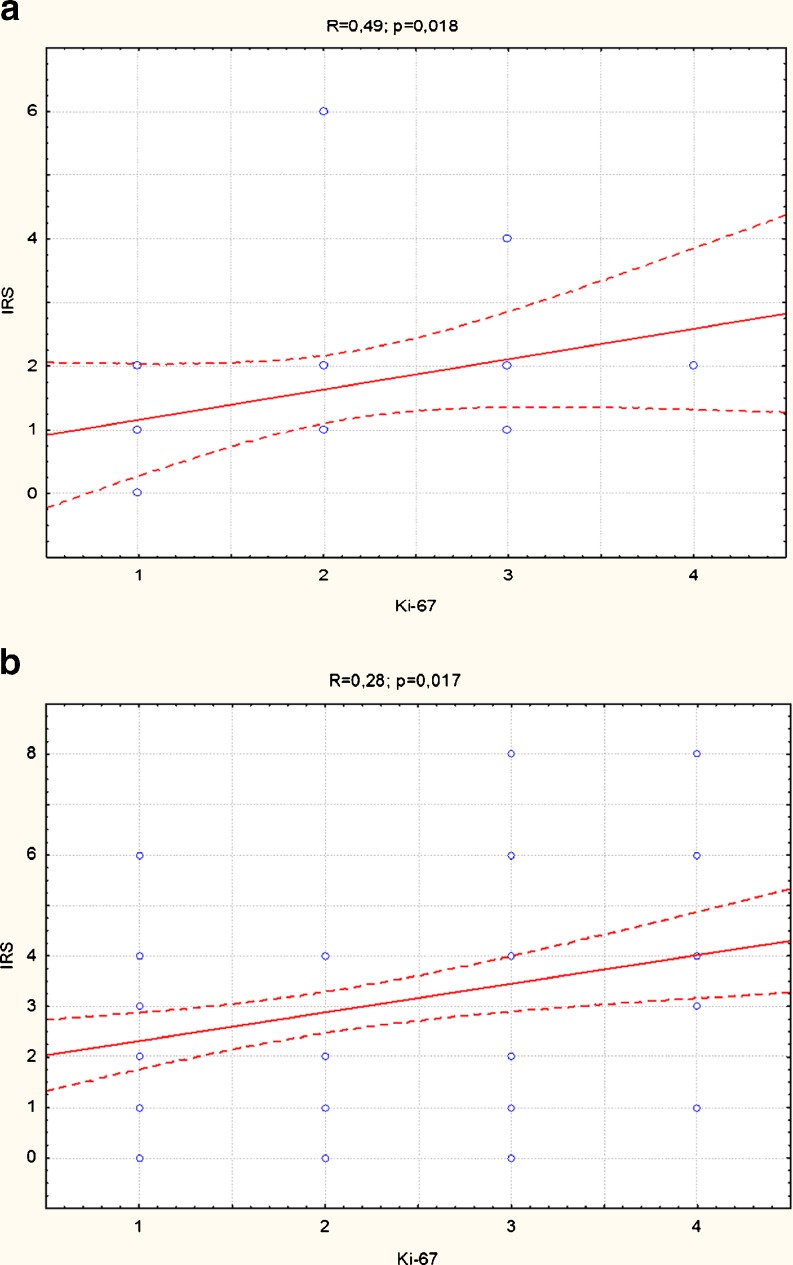
**a** Positive correlation between MT and Ki-67 antigen expression in AK. **b** Positive correlation between MT and Ki-67 antigen expression in SCC

The positive correlation between expression of MT and the expression of Ki-67 antigen was found in SCC as well as in AK, however the correlation with an AK lesions was stronger than with SCC (*R* = 0.28; *p* = 0.017 and *R* = 0.49; *p* = 0.018, respectively) (Fig. 3a, b). Such a phenomenon was absent in normal skin (*R* = −0.07; *p* = 0.76). The mentioned above weak correlation between MT and Ki-67 in SCCs had its reflection in relationships with Broders’ grading (G) of histological differentiation of SCC. Contradictory to significant positive correlation between Ki-67 antigen and the tumour’s G (*R* = 0.4; *p* < 0.001), the MT-expression revealed only weak correlation with tendency to increase in poorly differentiated SCC (*R* = 0.2; *p* = 0.06).

Moreover, various clinico-pathological variables, including age, gender of patients, localization, morphology and size of the lesions, as well as the depth of neoplastic infiltration and the density and depth of inflammatory infiltration did not affect MT-expression in both SCC and AK.

## Discussion

Only a few studies have been conducted to investigate the localization and the expression of MT in normal and neoplastic keratinocytes. It has been reported that in healthy skin MT are rather poorly expressed and restricted to basal and suprabasal layers [5, 32, 33] what was confirmed also in the present study. The same situation occurred in most AK lesions—MT expression was observed only in basal and spinous layers. In well-differentiated SCC the MT were detected predominantly in the periphery of tumor nest, while in poorly differentiated squamous cell carcinomas MT positive cells were dispersed throughout the entire tumor. Similar pattern of MT immunostaining has been noticed by other researches in SCC of skin and tongue [34, 35]. Additionally, head and neck cancers as well as breast adenocarcinomas have shown MT expression in healthy tissues surrounding the tumors [36, 37]. In the epithelium adjacent to SCC, the intense MT labeling of suprabasal layer was detected, what suggests that migrating tumor cells may induce MT-synthesis in healthy tissue adjacent to the tumor [34, 36].

It has been found, that epidermal MT-expression is independent on clinico-pathological factors like age and gender of patients, duration of the disease and size, type or localization of cutaneous SCC. In addition the depth of neoplastic infiltration as well as density and depth of inflammatory infiltration around SCC did not influence MT-expression. Similar observations were made by Borges et al. in non-melanoma skin cancers (NMSC) [34]. In their study no significant correlation between MT-expression and size of tumor, time of the disease or age of patients has been found either [34].

Various tumor parameters have been studied to predict the course of SCC. The worse prognosis was linked to: tumor size (T > 2 cm), type of precancerous lesion, localization, ulceration, immunosuppression, tumor thickness (>6 mm), G grading, the depth of tumor infiltration according to Clark's scale, histological tumor type and perineural invasion [30, 38, 39]. Some laboratory based methods were found to be useful as well. The invasiveness of SCC (and BCC) was correlated with level of MMP-1 (matrix metalloproteinase 1) and adhesion molecule CD44v6 [40]. Goldman et al. [38] demonstrated that a combination of tumor thickness and the expression of cathepsin D may have a high predictive value. It was also noticed that the differentiation of SCC was correlated with Ki-67 index [40]. In this study the grade of histological differentiation correlated positively with Ki-67 antigen in SCC and did not correlate with MT-expression, however the tendency to an increase of MT-expression in poorly differentiated SCC was observed. Perhaps the analysis of a larger group of patients would bring unequivocal finding and MT-expression would become the next good marker of SCC malignancy. Similar observation was made by Muramatsu et al. [37] in oral and pharyngeal SCC, where MT positive cell index was adequately higher in poorly differentiated SCC than in well and moderately differentiated SCC.

MT are engaged in cells proliferation and differentiation. In present study a positive correlation between MT-expression and Ki-67 expression has been described in AK and in SCC, which may testify the participation of both proteins in intense neoplastic proliferation of keratinocytes. The same correlation was found in colon adenocarcinoma and nosopharyngeal carcinoma [41, 42].

Many studies have proved that MT- expression is higher in cancers in comparison to precancerous lesions. It was observed in laryngeal and nosopharyngeal carcinomas [42, 43] and may result from higher demand for endogenous zinc and higher proliferative activity of the neoplastic cells. In the current reserach the MT- expression was also significantly higher in SCC than in AK and higher than in normal skin. Moreover, it has been described by other authors, that a higher MT-expression in NMSC is associated with tumoral aggressiveness. Borges et al. [34] analyzed the differences in MT- expression in BCC and SCC. In basal cell carcinomas MT-expression was observed in 18.5 % ± 21.2 % of tumor cells, while for squamous cell carcinomas immunostaining indices were 69.1 ± 14.4 % [34]. Similar conclusion was by Rossen et al. [44], who have examined MT-expression in different types of BCC and found that the immunoreactivity was high in infiltrating/morphea-like BCC, while most nodular BCC, which have less aggressive clinical behaviour, showed decreased or absent of MT-immunostaining [44].

The knowledge of the mechanism responsible for neoplastic transformation of keratinocytes gives an opportunity to create new diagnostic and therapeutic methods. In our study we have observed a high expression of Ki-67 antigen in AK and SCC as well, while MT-expression was statistically higher in SCC in comparison to its in AK lesions. A confrontation of those two values could serve as a helpful diagnostic method for differentiation of SCC from AK in doubtful cases.

To the best of our knowledge, our study is the first one comparing the MT- expression in cutaneous SCC and AK. Moreover, for the first time we showed correlation between expression of MT and Ki-67 antigen in cutaneous SCC and AK. In summary, based on our own results, it seems that MT-overexpression in SCC is an unfavorable factor promoting cutaneous carcinogenesis. This theory requires further investigations. Perhaps the antioxidative function and anti-immunosuppressive effect of metallothioneins prevail over their proliferative and anti-apoptotic properties in healthy skin, while MT-overexpression in neoplastic epidermal keratinocytes may lead to worsening course of the disease.
